# Phage–Bacterial Interaction Alters Phenotypes Associated with Virulence in *Acinetobacter baumannii*

**DOI:** 10.3390/v16050743

**Published:** 2024-05-08

**Authors:** Greater Kayode Oyejobi, Xiaoxu Zhang, Dongyan Xiong, Heng Xue, Mengjuan Shi, Hang Yang, Hongping Wei

**Affiliations:** 1 Key Laboratory of Special Pathogens and Biosafety, Center for Biosafety Mega-Science, Wuhan Institute of Virology, Chinese Academy of Sciences, Wuhan 430071, China; greateroyejobi@whu.edu.cn (G.K.O.); zxxwhiov@163.com (X.Z.); xiongdongyan18@mails.ucas.ac.cn (D.X.); xueheng979899@163.com (H.X.); shimengjuan20@mails.ucas.ac.cn (M.S.); 2 University of Chinese Academy of Sciences, Beijing 100049, China; 3 Key Laboratory of Combinatorial Biosynthesis and Drug Discovery (Ministry of Education), School of Pharmaceutical Sciences, Wuhan University, Wuhan 430072, China

**Keywords:** bacteriophages, *Acinetobacter baumannii*, phage resistance, phage–host interactions, fitness costs, virulence, phage therapy

## Abstract

Bacteriophages exert strong selection on their bacterial hosts to evolve resistance. At the same time, the fitness costs on bacteria following phage resistance may change their virulence, which may affect the therapeutic outcomes of phage therapy. In this study, we set out to assess the costs of phage resistance on the *in vitro* virulence of priority 1 nosocomial pathogenic bacterium, *Acinetobacter baumannii*. By subjecting phage-resistant variant Ev5-WHG of *A. baumannii* WHG40004 to several *in vitro* virulence profiles, we found that its resistance to phage is associated with reduced fitness in host microenvironments. Also, the mutant exhibited impaired adhesion and invasion to mammalian cells, as well as increased susceptibility to macrophage phagocytosis. Furthermore, the whole-genome sequencing of the mutant revealed that there exist multiple mutations which may play a role in phage resistance and altered virulence. Altogether, this study demonstrates that resistance to phage can significantly alter phenotypes associated with virulence in *Acinetobacter baumannii*.

## 1. Introduction

Bacteriophages (phages) can influence the evolution and diversity of bacterial communities, via varied coevolutionary mechanisms [1,2,3]. Phages engage their bacterial hosts in evolutionary interactions resulting in fluctuating selection dynamics over long periods [4,5]. During these arms race dynamics, phages exert selective pressure allowing the proliferation of bacterial strains presenting with different fitness costs. These costs can be explained because bacteriophages use structures present on the bacterial surface as entry receptors [6]; thus, strains with alterations in these receptors will be resistant to phage infection and may also exhibit either increased or reduced fitness costs in such features as virulence, antimicrobial susceptibility, growth rate, and so on [7,8].

Virulence, the measure of the pathogenicity of an organism, results from complex pathogen–host interactions and is of central importance to health [9]. Thus, some studies have attempted to study the role of ecological and evolutionary pressures from phages in limiting or increasing the virulence of bacterial pathogens [10,11,12,13]. These studies are especially important given the renewed interest in the use of phages in combating the increasing crisis of antimicrobial resistance. Although some case reports have shown successes in experimental phage therapy as compassionate use (reviewed in [14]), clinical trials to prove its credibility and safety have failed [15,16,17]. Thus, information regarding phage–host interactions that affect bacterial virulence under different conditions might be helpful in knowing where we stand with regard to the inclusion of phage therapy in modern medicine.

Phage resistance can alter bacterial virulence due to the loss of important virulence determinants that have a dual function as phage receptors [18]. These structures include flagella, pilus, LPS, and capsule [1]. Mutations in genes encoding these structures often lead to phage resistance and altered virulence [6]. Data from existing studies have shown variations in virulence costs following phage resistance; while some have reported enhanced virulence, some others have reported decreased virulence [10,19,20]. Therefore, the complexity of this phenomenon is worth researching. Strain-to-strain variations in fitness costs on bacterial virulence following the evolution of phage resistance may be critical in infection outcome and the success of phage therapy. In the current study, our model bacterium is carbapenem-resistant *Acinetobacter baumannii* (CRAB), a pathogenic bacterium considered as priority 1 on the list of World Health Organization (WHO) priority pathogens for research and development of new antibiotics [21], and an urgent public health threat by the Centers for Disease Control and Prevention [22].

Previously, we experimentally adapted *A. baumannii* WHG40004 to a lytic phage P21, and thereafter, isolated a phage-resistant bacterial mutant, named Ev5-WHG [23]. In the current study, we subjected Ev5-WHG, alongside the wild-type strain to *in vitro* experiments with the aim of understanding the correlation between the evolution of phage resistance and fitness costs on phenotypes associated with bacterial virulence. Overall, we found that phage–bacterial interaction significantly altered certain phenotypes associated with virulence in phage-resistant *Acinetobacter baumannii*.

## 2. Materials and Methods

### 2.1. Bacterial Culture and Phage-Resistant A. baumannii Mutant

*A. baumannii* strain WHG40004 was obtained from bacterial culture stocks of the Diagnostic Microbiology Unit of the Wuhan Institute of Virology, CAS, P.R. China [24]. Phage-resistant strain Ev5-WHG was previously obtained through a 10-day bacterial evolutionary response to phage [23]. Bacteria was grown in lysogeny broth (LB) medium and overnight culture incubation was performed at 180 rpm at 37 °C. To prevent the potential influence of the difference in bacterial concentration as a result of a possible different growth rate between ancestral bacterial strain and phage-resistant mutant, we chose to normalize all of the assay measurements for each isolate by the isolate’s level of growth (OD_600_).

### 2.2. Hydrogen Peroxide Bacterial Killing Assay

Bacteria was cultured in LB. After 24 h, bacteria were washed twice in PBS, and diluted to a concentration of 2.5 × 10^6^ colony forming units (CFU) per 250 μL reaction mixture in a 2 mL Eppendorf tube. Hydrogen peroxide (H_2_O_2_) was added to a 0.1% final concentration and the tubes were incubated for 30 min at 37 °C with shaking at 250 rpm. The reaction was stopped by the addition of 0.2% 1000 U/mL exogenous catalase (Yuanye Bio-Technology, Shanghai, China; CAS S25070). Bacterial viability was assessed by dilutions on LB plates for the enumeration of surviving CFUs.

### 2.3. Bacterial Serum Killing Assay

Overnight cultures of bacteria were centrifuged and suspended in sterile PBS to generate a suspension of 1 × 10^7^ CFU/mL. Serum collected from healthy volunteers were used for this assay. To obtain heat-inactivated serum, serum was treated for 30 min at 56 °C. Serum (450 μL) was transferred into sterile tubes and mixed with 50 μL bacterial sample, which resulted in 1 × 10^6^ CFU/mL. The tubes were incubated at 37 °C for 6 h with shaking (250 rpm), and then dilutions were plated on a LB agar plate for the enumeration of surviving CFUs.

### 2.4. Cytotoxicity Assay

Monolayers of A549 cells were infected with bacteria at a MOI of 100. At 5 h post-infection, supernatants of uninfected or infected cells were used to quantify the activity of the cytosolic enzyme lactate dehydrogenase (LDH; Abbkine Inc., Beijing, China). A positive control supplied with the LDH Cytotoxicity Assay Kit (LDH; Abbkine Inc., China) was used. The percentage of LDH activity was determined using the following formula: percentage of release = (experimental LDH release − spontaneous LDH release)/(maximal LDH release − spontaneous LDH release) × 100% [25].

### 2.5. Cell Culture Conditions

A549 cells were cultured in Dulbecco’s modified Eagle medium: nutrient mixture F-12 (DMEM/F12, Gibco, Shanghai, China) supplemented with 10% fetal bovine serum (FBS, Sigma-Aldrich, Shanghai, China). Macrophage RAW264.7 cells were cultured in DMEM (Gibco, Shanghai, China) supplemented with 10% FBS. All cells were cultured in a humidified atmosphere of 5% CO_2_ at 37 °C.

### 2.6. Bacterial Adhesion to Epithelial Cells

A549 epithelial cells were seeded in 48-well plates in DMEM/F-12 complemented with 10% FBS at 37 °C with 5% CO_2_. The cells were infected with bacteria at an MOI of 10 and incubated at 37 °C with 5% CO_2_. Two experimental treatments were set up. In one treatment: after 3 h of incubation, epithelial cells were washed with sterile PBS and disrupted with 0.025% Triton X-100 to enumerate total bacteria in each well (N_t_). In the other treatment: after 3 h of incubation, epithelial cells were washed thrice with sterile PBS and resuspended in DMEM/F-12 complemented with 10% FBS and 1% antibiotics (200 mg/mL gentamycin and 10 mg/mL penicillin) to kill unattached bacteria and further incubated for 1 h at 37 °C with 5% CO_2_. The amount of viable intracellular bacteria was determined (N_i_). The bacteria adhered on the cell surface (N_a_) was expressed as the difference between the total bacteria and internalized ones (N_a_ = N_t_ − N_i_) [26].

### 2.7. Bacterial Macrophage Phagocytosis and Killing Assay

RAW264.7 macrophages were seeded in 48-well plates in DMEM complemented with 10% FBS at 37 °C with 5% CO_2_. The cells were infected with bacteria at a MOI of 10 and incubated at 37 °C with 5% CO_2_. After 4 h of incubation, supernatants were collected and cells washed three times with sterile PBS and disrupted with 0.025% Triton X-100 to release intracellular bacteria. The amount of viable intracellular bacteria in both the supernatants and macrophages was determined by plating on LB agar plates and incubation overnight at 37 °C. Macrophage–bacteria interaction for 4 h was used to assess bacterial survival in macrophages [27].

### 2.8. Swarming Motility Test

Motility agar was prepared using LB plus 0.5% agar and then inoculated with 1 μL of overnight liquid culture on the agar surface. Plates were incubated at 37 °C, and the diameter of the motility zone around the point of inoculation was measured on the plates after 20 h of incubation.

### 2.9. Statistics

Data are presented as the means ± standard error of mean (SEM). Differences in quantitative measurements, including epithelial adhesion and internalization assays, and macrophage assay, were assessed by Student’s *t* test or one- or two-way analysis of variance (ANOVA). Differences were considered significant when *p* < 0.05.

## 3. Results

### 3.1. Ev5-WHG Showed Reduced Performance in Host Microenvironments

**Figure 1:**
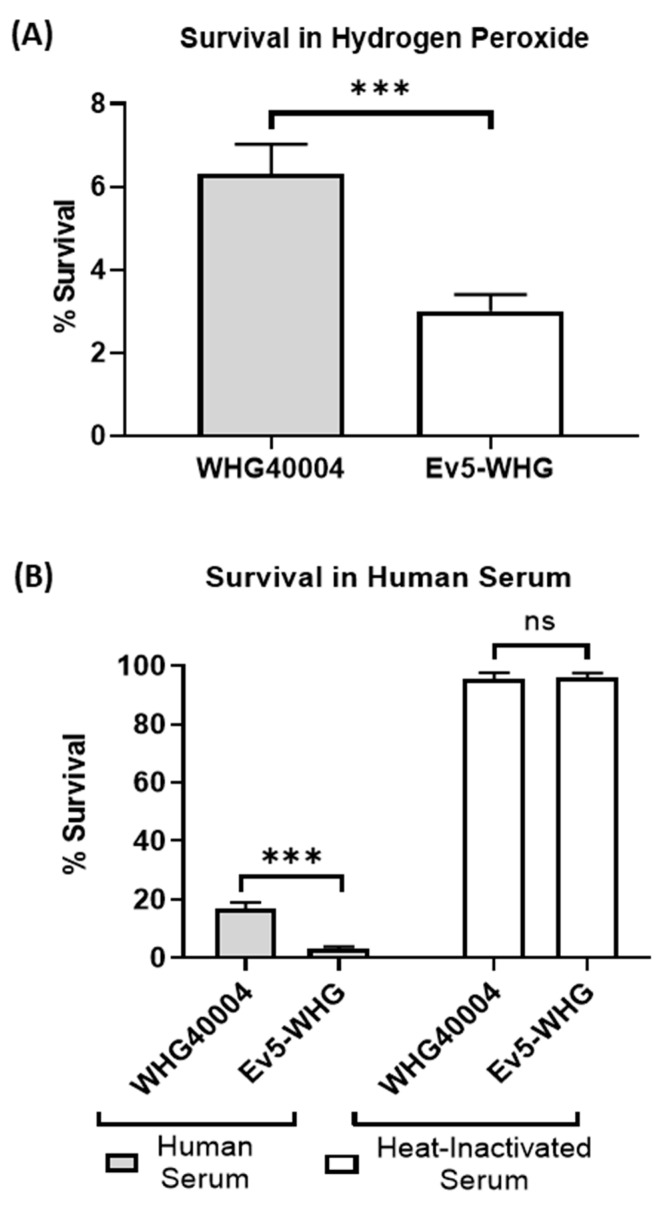
Performance in a host microenvironment. (**A**) Percentage of surviving bacteria following treatment with hydrogen peroxide. Data are expressed as mean ± SEM (n = 3). ***: *p* < 0.001; (**B**) Percentage of surviving bacteria in normal human serum. Data are expressed as mean ± SEM (n = 4). ***: *p* < 0.001; ns: not significant. Presented results are an average of 3 or more independent experiments.

We tested the growth ability of Ev5-WHG in media to simulate the host microenvironment—serum and hydrogen peroxide. The Ev5-WHG mutant and WHG40004 were compared by measuring their abilities to resist the loss of viability and inhibition of bacterial metabolism following exposure to killing by hydrogen peroxide. Results revealed that Ev5-WHG displayed lower resistance than wildtype bacteria (Figure 1A). Also, we tested the survival of both bacteria to healthy human serum activities. Results (Figure 1B) showed that the Ev5-WHG mutant had increased susceptibility to killing by human serum, which was due to complement activities, as shown by the results of bacterial survival in heat-inactivated serum.

### 3.2. Ev5-WHG Was Less Cytotoxic on A549 Epithelial Cells

**Figure 2:**
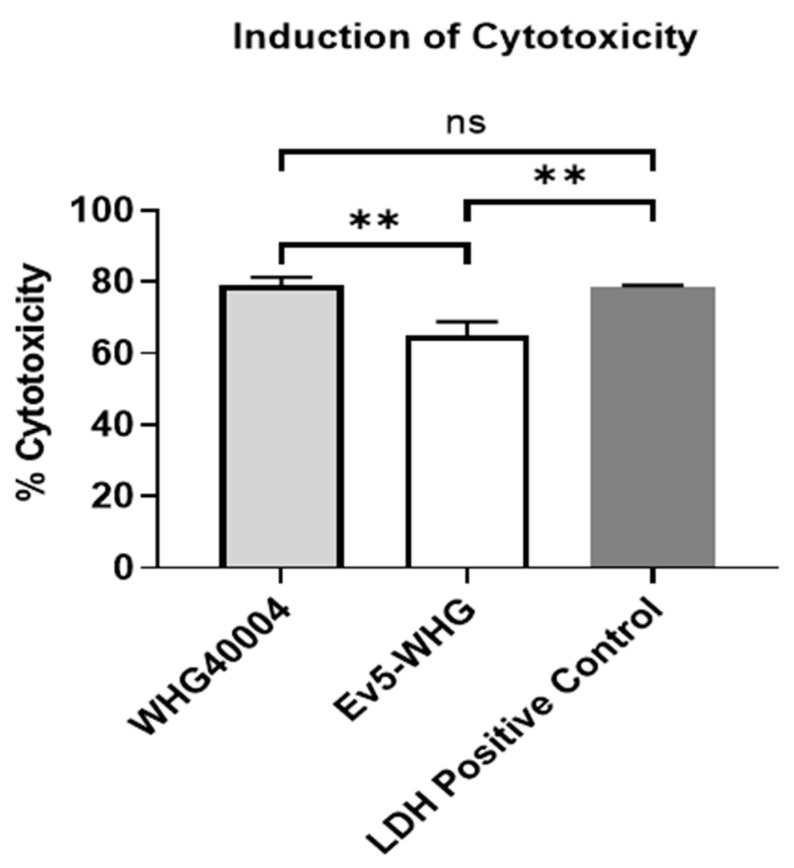
Cytotoxicity induction by bacteria on A549 cells. Data are expressed as mean ± SEM (n = 3). **: *p* < 0.01; ns: not significant. Presented results are an average of 3 or more independent experiments.

We compared the cytotoxicity of WHG40004 and Ev5-WHG on A549 cells (Supplementary Materials). By reading the absorbance and calculating the percentage cytotoxicity, it was discovered that resistant mutant Ev5-WHG was less cytotoxic than wildtype bacteria. Results showed that while WHG40004 induced ~79%, Ev5-WHG induced a significantly lower cytotoxicity of ~65% (Figure 2).

### 3.3. Ev5-WHG Displayed Impaired Adhesion and Invasion to A549 Epithelial Cells, and Decreased Resistance to Killing by RAW264.7 Macrophage Cells

**Figure 3:**
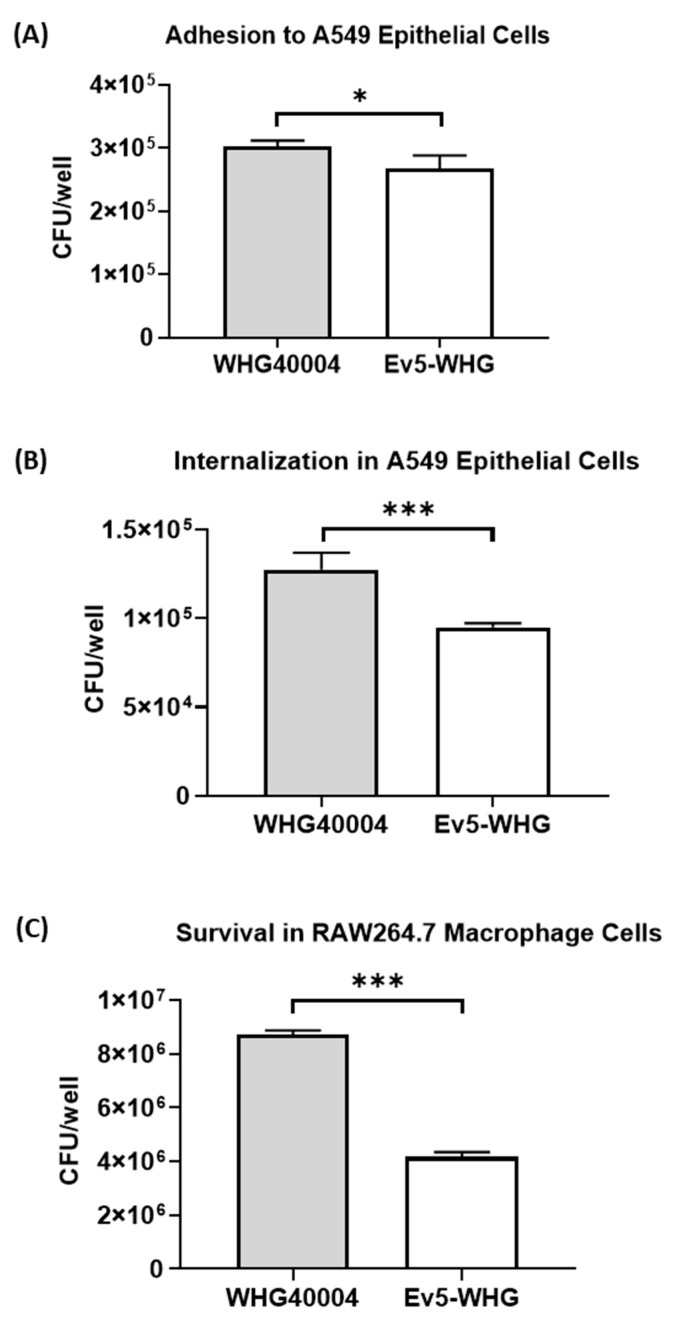
Performance in mammalian cell lines. (**A**) Bacterial adhesion to A549 epithelial cells. Data are expressed as mean ± SEM (n = 4). *: *p* < 0.1; (**B**) Internalization of bacteria in A549 epithelial cells. Data are expressed as mean ± SEM (n = 4). ***: *p* < 0.001; (**C**) Bacterial survival in RAW264.7 macrophage cells. Data are expressed as mean ± SEM (n = 4). ***: *p* < 0.001. Presented results are the averages of 3 or more independent experiments.

We subjected the phage-resistant mutant alongside the wildtype stain to interaction with monolayers of A549 cells, and then assessed their abilities to adhere to and invade these cells. The results indicated that phage-resistant mutant Ev5-WHG had impaired ability to adhere to epithelial cells in comparison with the wildtype strain (Figure 3A). Furthermore, the mutant had a significantly reduced ability to invade A549 epithelial cells (Figure 3B). We also measured and compared the extent to which the mutant Ev5-WHG and wildtype strain can resist macrophage phagocytosis and killing *in vitro* in RAW264.7 cells. We found that Ev5-WHG had a lower resistance to killing by macrophage (Figure 3C).

### 3.4. Ev5-WHG Displayed Similar Motility to Wildtype Strain

**Figure 4:**
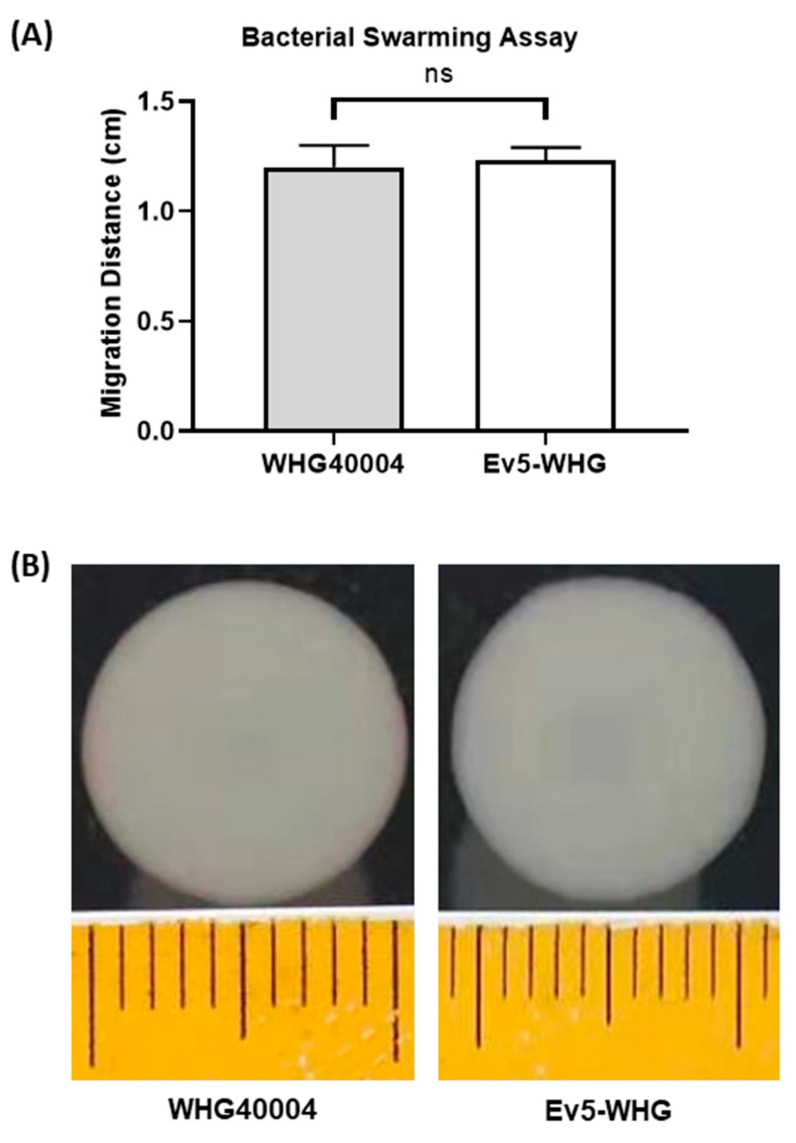
(**A**,**B**). Bacterial swarming assay showing no difference in bacterial migration distance between mutant and WT bacteria. Data are expressed as mean ± SEM (n = 2). ns: not significant.

We thought that a lower invasion of mammalian cells could have something to do with a change in motility. To rule out this possibility, we compared the motilities of mutant Ev5-WHG and wildtype bacteria. However, by comparing the bacterial migration distance, we observed that Ev5-WHG displayed similar motility to the wildtype strain (Figure 4).

### 3.5. Multiple Mutations in Ev5-WHG May Play Role in Phage Resistance and Altered Virulence Phenotypes

We previously performed whole-genome sequencing on resistant mutant and wildtype bacteria [23]. Genomic analysis revealed that there was a total of 22 single nucleotide mutations on 13 genes in the genome of the resistant mutant [23]. Although this remains to be verified, these mutations may play important roles in both phage resistance and the observed alteration in virulence phenotypes.

## 4. Discussion and Conclusions

Antibiotic resistance in *A. baumannii* is a problem [28], necessitating the need for an alternative approach in the treatment of its infections. The use of bacteriophages is a promising alternative. However, as natural partners, bacteriophages exert strong selection on their bacterial hosts to evolve resistance. In the current study, we found that the fitness cost of phage-resistant *A. baumannii* decreased its performance in mammalian cell lines.

The phage-resistant mutant in this study displayed less internalization in model human alveolar basal epithelial cells and reduced resistance to phagocytosis by model murine macrophage cells (Figure 3B,C). The adherence and invasion of epithelial cells play roles in the successful establishment of infection by bacterial pathogens and are critical determinants of bacterial virulence [29]. The role of macrophages in *A. baumannii* infection has been described, and they are known to play a crucial role in early host defense, especially against respiratory *A. baumannii* infection [30]. Macrophages phagocytose *A. baumannii* and kill them with reactive oxygen species (ROS) and nitric oxide (NO) in the early stage of inflammatory responses in respiratory *A. baumannii* infections [30,31,32]. Also, Ev5-WHG displayed lower resistance to killing by hydrogen peroxide (Figure 1A). Hydrogen peroxide (H_2_O_2_) is produced by inflammatory and vascular cells and induces oxidative stress, which may contribute to vascular disease and endothelial cell dysfunction [33]. We infer that Ev5-WHG suffered a reduced ability to survive under oxidative stress induced by H_2_O_2_. This may reveal an interesting physiological property of *A. baumannii* regarding its amino acid uptake system, implicating this system as important determinant of virulence.

Furthermore, Ev5-WHG in this study exhibited a reduced ability to survive in human serum (Figure 1B). Results indicate that killing was dependent on complement activity because no reductions in viability were observed with heat-inactivated serum. The increased complement susceptibility of Ev5-WHG is indicative of the reduced virulence potential.

Following whole-genome sequencing and genome analyses, phage-resistant mutant in this study was previously found to contain 22 single nucleotide mutations on 13 genes [23], which could have contributed to phage resistance in Ev5-WHG. Furthermore, scanning electron microscopy (SEM) image analysis revealed that the cell surface appearance of the Ev5-WHG mutant was rough and appeared thickened compared to a smooth appearance in wild-type bacteria [23]. Also, phage adsorption assays showed that P21 had impaired attachment to Ev5-WHG [23]. Put together, these results suggests that the change in cell surface appearance could have probably contributed to the observed differences in the current study. Available literatures [34,35] have shown that mechanisms relating phage resistance with fitness costs on the virulence of *A. baumannii* seem to have been limited to surface modifications. For example, Altamirano et al. [34] previously showed that phage-resistant *A. baumannii* mutants harbored loss-of-function mutations in genes responsible for the biosynthesis of capsular polysaccharides, and as a result, exhibited a reduction in biofilm formation, resensitization to antibiotics, human complement and phages, and a reduction in fitness in a mouse model of bacteremia. Also, Wang et al. [35] showed that phage-resistant *A. baumannii* strains displayed mutations in genes that alter the architecture of the bacterial envelope at the capsule and the outer membrane levels, and consequently displayed reduced virulence in a *Galleria mellonella* infection model, as well as an increased sensitivity to colistin. In the current study, we could not verify the impacts of the resulting mutations on the observed phenotypic changes in phage-resistant bacteria. More studies are needed to confirm the roles of these mutations in linking phage resistance with virulence in *A. baumannii*.

Bacterial virulence is a complicated phenomenon, and only in some few cases is it determined by a single factor. *In vivo* virulence was not tested for in this study and could have obscured some effect that was not detected in this study. More studies to confirm and investigate the molecular mechanisms by which the observed mutation in this study contributed to the decreased performance and inferred the reduced virulence of phage-resistant mutant are necessary. Characterizing the proteins involved in these mechanisms and *in vivo* experiments could help to better understand the relationship between phage resistance and virulence in *A. baumannii*. It is worth noting that different mechanisms may be involved in the fitness costs of phage-resistant *A. baumannii*, as shown in previous studies [34,35]. Therefore, fitness costs are thought to be context-dependent [36], and it is difficult to generalize a resulting reduced virulence in phage-resistant *A. baumannii* mutants. More studies are needed to determine the virulence costs on mutants under different conditions.

Conclusively, our study demonstrates a relationship between phage resistance and bacterial performance in activities that relate to virulence. Altogether, our observations suggest a potential reduced virulence in the phage-resistant mutant in this study, providing yet another window of opportunity in combating *A. baumannii*.

## Data Availability

The sequenced genomics data of bacteria used in this study have been deposited in the Genome Warehouse in the National Genomics Data Center (https://ngdc.cncb.ac.cn/gwh) [26], Beijing Institute of Genomics (China National Center for Bioinformation), Chinese Academy of Sciences. The sequenced data was accessed on 21 December 2021. The BioProject accession number is PRJCA007903. The accession numbers of the submitted bacterial genome is GWHBHAO01000000, and sequencing data can be accessed on https://ngdc.cncb.ac.cn/search/?dbId=gwh&q=GWHBHAO01000000.

## Supplementary Materials

The following supporting information can be downloaded at: https://www.mdpi.com/article/10.3390/v16050743/s1, Figure S1: Bacterial swarming assay showing no difference in bacterial migration distance between mutant and WT bacteria; Figure S2: Growth curves of WHG40004 and Ev5-WHG.
