# Supplementary material for: Phage–Bacterial Interaction Alters Phenotypes Associated with Virulence in Acinetobacter baumannii
Source: Viruses. 2024 May 8;16(5):743. doi: 10.3390/v16050743 (PMC11125765; doi:10.3390/v16050743)
Supplement: Supplementary file 1 [file viruses-16-00743-s001.zip › Supplemental Material.pdf]

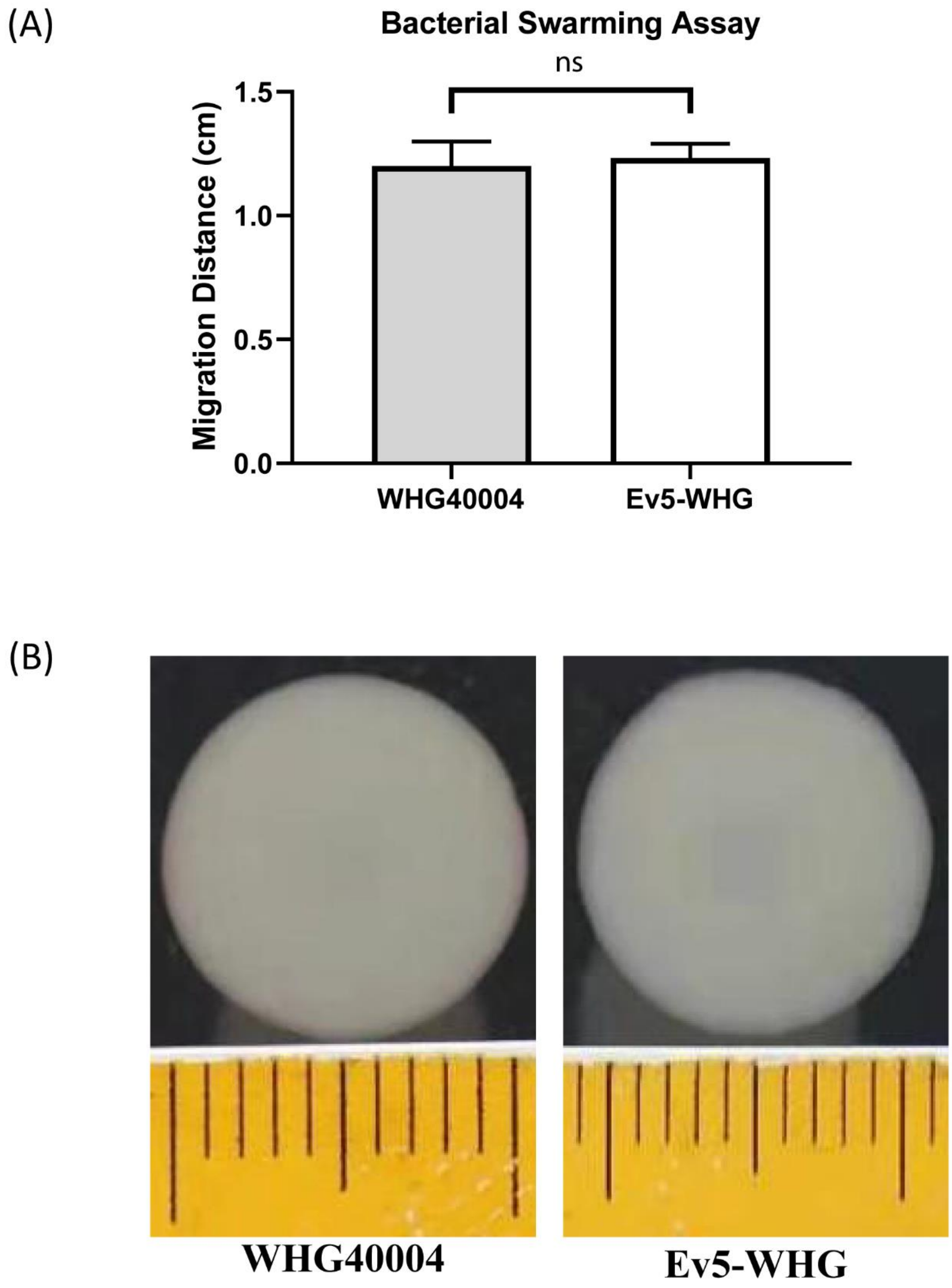

Figure S1A-B: Bacterial swarming assay showing no difference in bacterial migration distance between mutant and WT bacteria. Data are expressed as mean ± SEM (n = 2). ns: not significant.

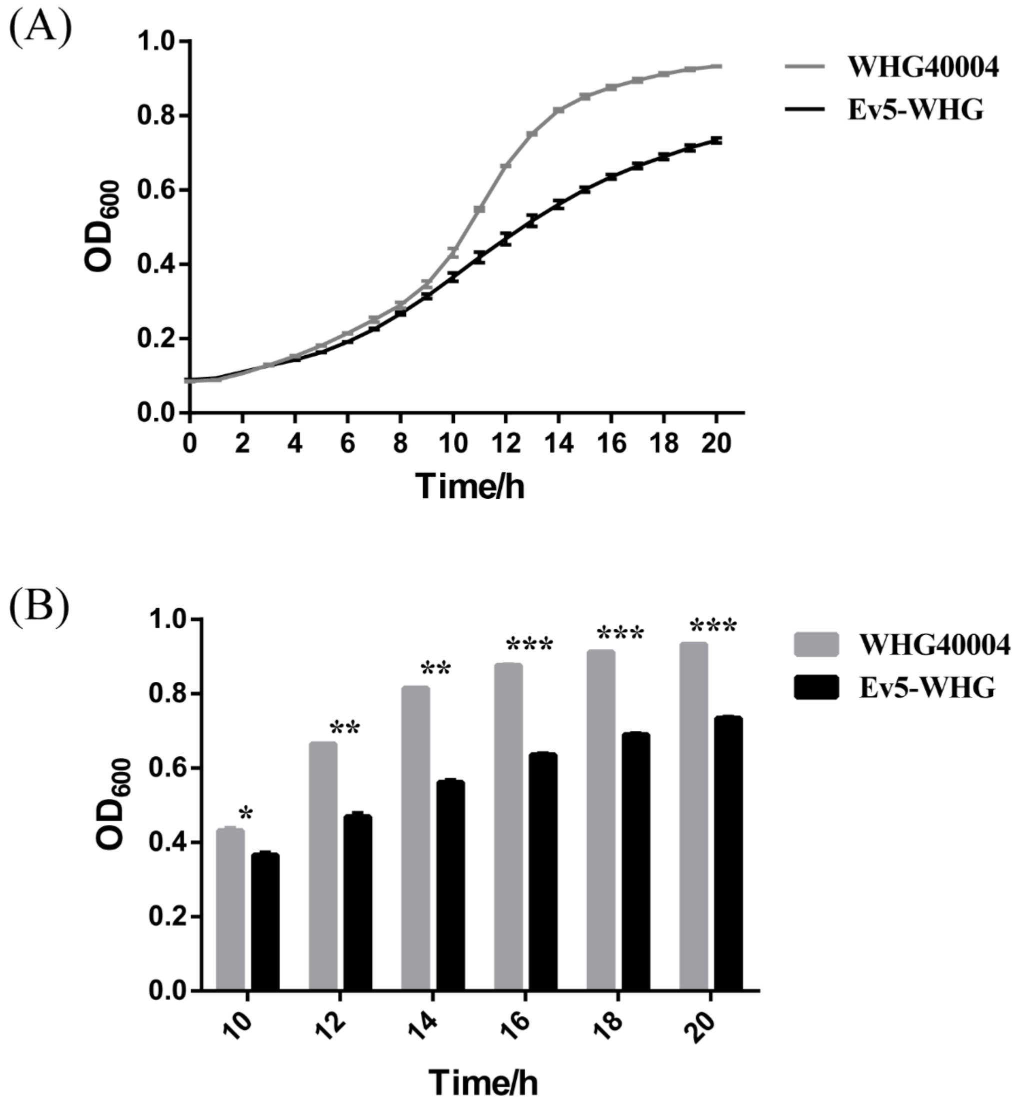

Figure S2: Growth curves of WHG40004 and Ev5-WHG. Error bars show average values and SD of three independent experiments. *: $p<0.1$; **: $p<0.01$; ***: $p<0.001$.
