# Supplementary figures and images for: Phage–Bacterial Interaction Alters Phenotypes Associated with Virulence in Acinetobacter baumannii
Source: Viruses. 2024 May 8;16(5):743. doi: 10.3390/v16050743 (PMC11125765; doi:10.3390/v16050743)

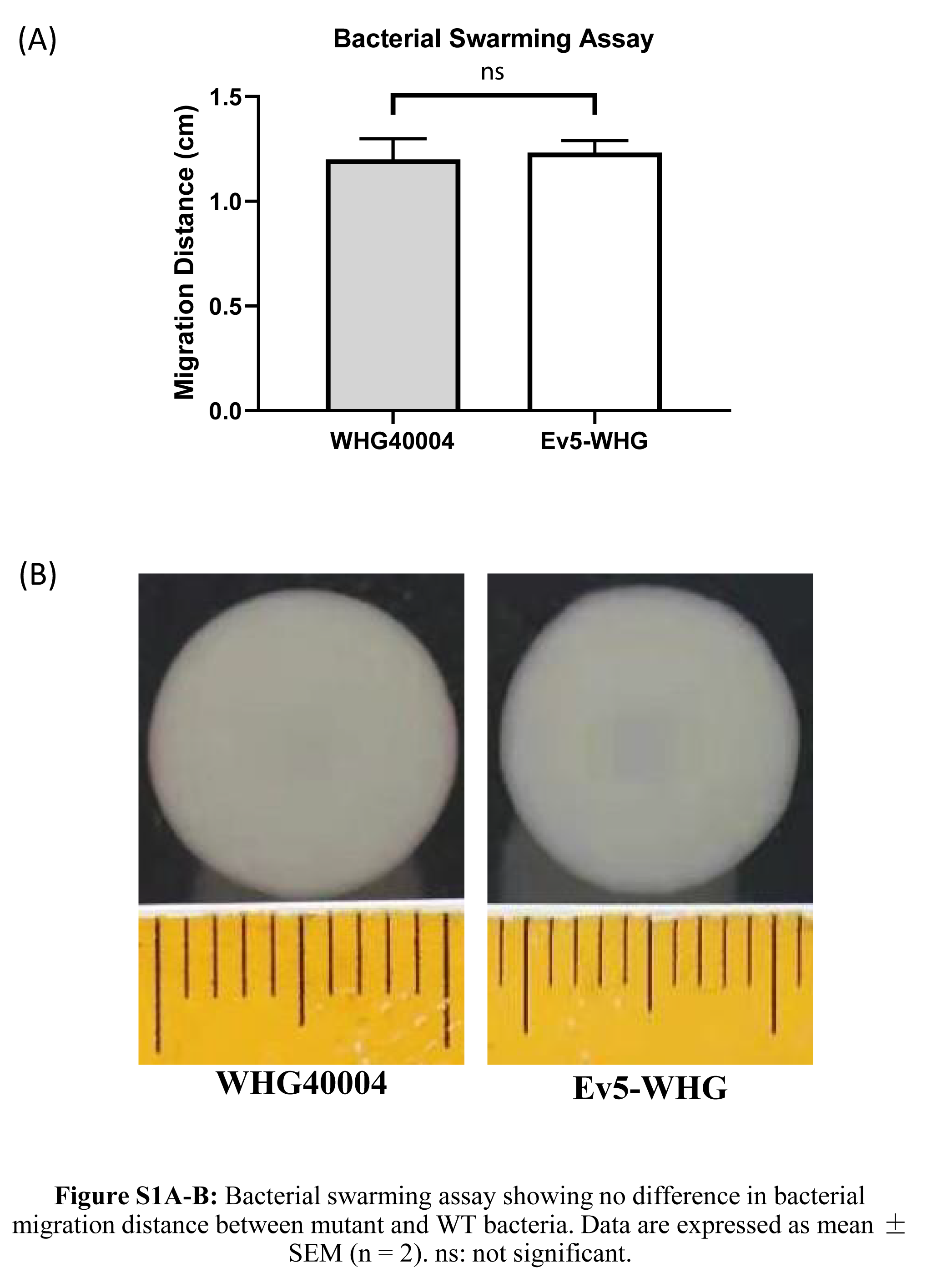

Supplement: Supplementary file 1 [file viruses-16-00743-s001.zip › Figure S1.tif]

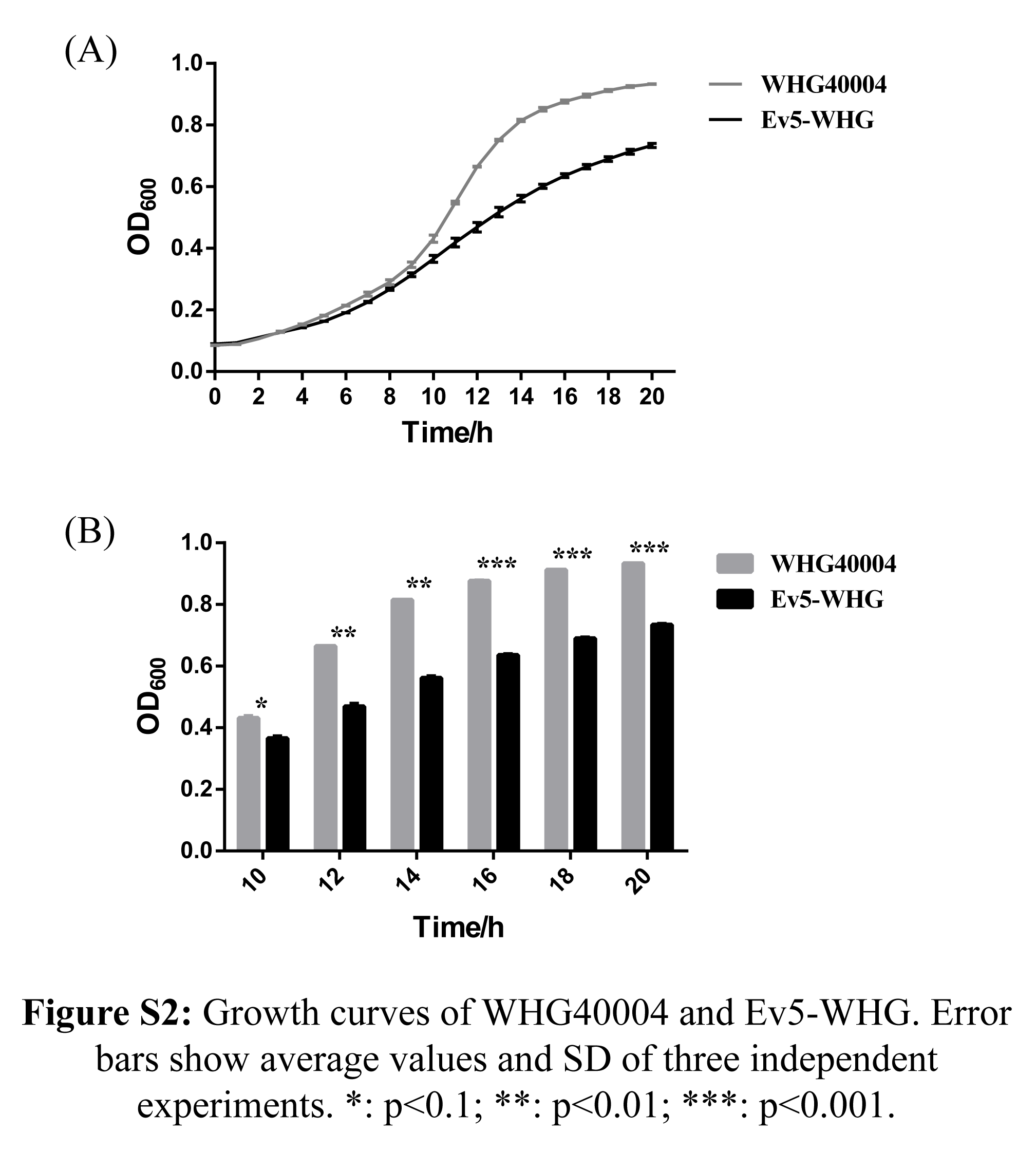

Supplement: Supplementary file 1 [file viruses-16-00743-s001.zip › Figure S2.tif]
